# The Week's Work

**Published:** 1910-08-06

**Authors:** 


### The Week's Work.

### THE ROYAL VISIT TO THE LONDON HOSPITAL.

King George has shown once more his practical interest in the charitable institutions, whose prime objects are the relief of sickness and distress, by visiting and personally inspecting the London Hospital on Saturday afternoon last. This visit, though of a strictly private nature, so far as the institution was concerned, was looked forward to, as it was the first public drive which their Majesties have permitted themselves through the Metropolis since the death of King Edward. It is significant that the occasion for this first public ceremonial should have been an errand of mercy, but those who know the eager interest which King George has constantly displayed in hospital work, will realise that last Saturday's visit was not, and is not likely in the future to be, an isolated mark of the King's appreciation of the work that the London and other hospitals are doing. The Royal interest, we hear, has already stimulated public generosity and rejuvenated general interest in the work of the East End hospital, while it is unquestionable that it will in the future beneficially affect the cause of charity in this country. His Majesty showed on Saturday that his appreciation was real and thorough, and hospital and medical workers will feel gratified at the spontaneous way in which it was expressed since they know that the King is a competent critic, who has spared no pains to become acquainted with the inner working of the hospitals. The incident related by King George on Saturday last shows very well that he did his best to become acquainted with the work that is being done in our Metropolitan institutions, and that he has not been satisfied with confidential reports, but has investigated matters for himself, adopting on one occasion at least, a ruse to obtain the necessary knowledge of internal arrangements upon which to base an opinion. Hospital workers will applaud the spirit that prompted these searching investigations, and everyone concerned with institutional work will appreciate the sincere commendation that the King's recognition of the import- and excellence of that work carries with it.

### HOSPITAL ABUSE.

The question of hospital abuse has lately been much discussed in America, and an interesting sidelight on the prevailing trend of institutional opinion on this important subject is afforded by one of the provisions of the new State Hospital Law for New York. This attempt at a uniform system of legislation for the hospitals, in many ways highly interesting because of the various matters with which it deals, is specially worth noticing since it is the evolution of certain definite suggestions furnished by institutional workers. The Bill was finally approved by the Governor, and was signed and became law about a month ago, so that there has really been no chance of seeing in what way its provisions will modify present schemes so far as in-patients are concerned. It provides for the proper inquiry, by the superintendent of a hospital, or by a person duly delegated by him for such purpose, into the means of every in-patient, and for the enforcing of the payment of such fees as the hospital authorities may exact in cases where it is found that the patient is in a position to pay. "In cases where the patient is unable to pay, or where his immediate relatives cannot afford to do so, the unpaid cost of his maintenance in hospital shall become a charge upon the town which maintains the institution in which he has been treated, unless he happens to be an outsider, in which case his expenses shall be borne by the State upon whose civil division he will become a "pauper charge." This is in accordance with the municipal regulations in Germany, and the system, so far as the large German hospitals are concerned, has on the whole worked admirably. In the New York Act, however, no provision, so far as we have been able to find, has been made for the payment of members of the hospital staff. Under the municipal regulations in Germany members of the staff are paid. It is true the annual salary is in many cases a small one, utterly out of proportion to the ability and the professional standing of the recipient, but it is at least an admission of the principle, and as such it is well to press for its recognition by law. The new Act is an interesting one, and hospital workers will watch its results with attention in the immediate future in view of the problem of hospital abuse which faces us in this country.

### HOSPITALS AND THE POST OFFICE.

Many years ago the suggestion was made that charitable institutions, admittedly doing excellent work, and of recognised standing, should be allowed certain privileges which would enable them to carry on their work more efficiently and successfully. It was pointed out that hospitals of necessity spend large amounts on postage. The larger the success of a voluntary hospital, the greater must be its stamp bill. Every energetic secretary makes the fullest possible use of the facilities which are given to the public by the Post Office in the way of telephoning or telegraphing, and it is not unreasonable to propose that, in the circumstances, he should be allowed to make use of these facilities at a lower charge. To take telegrams, for instance, the Press, which is by no means a charitable institution, is favoured by the telegraph service, and although hitherto no one has suggested that hospitals should have free telephones or that hospital superintendents should be allowed to frank their letters—a privilege which would doubtless give rise, if it were granted, to the same abuses which led to the abolition of the Parliamentary system of franking—is it so unreasonable to hope that the Post Office will in the near future see its way clear to introduce a "hospital rate" for telegrams, much on the same lines as it has admitted a Press rate for newspaper wires? At present it is against such an innovation. Mr. G. W. Dick, of Leith, who has interested himself in this matter to the extent of making a personal appeal to the Postmaster-General, asking him to agree to a reduced scale of charges for hospitals and other recognised charitable institutions, has received a reply to the effect that the suggestion has been carefully considered "but the Postmaster-General regrets that he does not see his way to adopt it." We are inclined to believe that if such a reduced tariff as Mr. Dick proposes were instituted, the revenue which the Post Office derives from hospitals would be likely to increase, but we are, of course, for the moment merely concerned with the institutional aspect of the question. Such a reduction would be of undoubted service to charitable organisations of all kinds, and it ought to be relatively easy to effect some arrangement whereby any abuse could be prevented. We trust Mr. Dick will not let the matter rest, but return to it again and again until he succeeds in changing the "regrettable" attitude of Mr. Samuel.

### THE MATRONSHIP OF ST. BARTHOLOMEW'S HOSPITAL.

We have already commented upon the appointment of Miss McIntosh as matron of St. Bartholomew's Hospital, and there is no necessity to go into the matter once more. But it is gratifying to be able to state that the Court of Governors has acted firmly in the matter, and refused to allow itself to be intimidated by a certain section of malcontents into reviewing the appointment. The grounds alleged for such review were utterly inadequate, as everyone who knows Miss McIntosh's career at the London knows well enough. Apart altogether from the fact that the agitation against the new matron was in the worst taste, and was carried on with a degree of acerbity which certainly did not reflect credit upon the protestants, it was an attempt to evoke a mutiny, and to break up the solidarity which is so necessary between the governing body and the staff. As such it was imperative that the Governors should have shown that they were firm and united in their decision, and the manner in which the subject was treated at the recent Court is therefore to be commended. It is sincerely to be hoped that no further attempts will be made to sow the seeds of discontent and ill-feeling, but that everyone who has at heart the interests of the oldest hospital in the Metropolis will loyally support the Governors and the new matron in their work on its behalf.

### THE DRUG MARKET.

Rather more interest has been taken in the drug market during the last few weeks, but business this week has naturally been somewhat quieter owing to the Bank Holiday. Opium continues to be the chief centre of interest, and prices are steadily decreasing in consequence of the large crop which is now being collected. Codeine has again been reduced 5d. per ounce, which is the third reduction (amounting in all to 1s. 3d. per ounce) during the last three weeks. Morphine is also lower, and the tendency is for prices to recede still further. Cape aloes is slightly lower in price. Castor oil is tending dearer. Buchu leaves are again very considerably dearer, having advanced fully 1s. 6d. per lb. Coca leaves are slightly higher in value. Columba root is decidedly dearer. Ipecacuanha is slightly cheaper. The price of jalap is well maintained. Rhubarb continues to sell at low rates. The demand for menthol has quietened down. The slight reduction in the price of quicksilver announced last week has not been followed by a reduction in the price of mercurial salts.

